# Supplementary material for: Accessing valley degree of freedom in bulk Tin(II) sulfide at room temperature
Source: Nat Commun. 2018 Apr 13;9:1455. doi: 10.1038/s41467-018-03897-3 (PMC5899090; doi:10.1038/s41467-018-03897-3)
Supplement: Supplementary file 1 — Supplementary Information [file 41467_2018_3897_MOESM1_ESM.pdf]

Supplementary information

Title: Accessing Valley Degree of Freedom in Bulk Tin(II) Sulfide at Room Temperature

Authors: Lin et al.

Supplementary Note 1. Using Raman peaks to define armchair and zigzag directions

The spectra obtained using the CCD detector consists of multiple peaks, which upon deconvolution yield important information about the angular dependence of the Raman modes (Fig. 1c). We note from previous work [1] that the Raman tensors of the four observable Raman modes, which reside at 95.5cm^{-1}, 190.7cm^{-1}, and 216.8cm^{-1} for A_g and 162.5cm^{-1} for B_{3g}, have only two (one) non-zero components that contributes to the polarization dependent Raman under excitation in the -z direction for the A_g (B_{3g}) mode. The equations provided by ref [1] are:

$$I_{A_{g,\parallel}} = B\cos^2(\theta) + C\sin^2(\theta) \tag{1}$$

$$I_{A_{g,\perp}} = \frac{(C-B)^2}{4}\sin^2(2\theta) \tag{2}$$

$$I_{B_{3g,\parallel}} = F^2\sin^2(2\theta) \tag{3}$$

$$I_{B_{3g,\perp}} = F^2\cos^2(2\theta) \tag{4}$$

In order to rigorously determine the armchair (y) direction of our samples, we utilized the listed equations to determine the sample orientation and fixed θ=0°.

We first begin with fitting the B_{3g} peak, primarily because of simplicity in handling the fitting constant, F. In the fitting equation, we define a phase offset ω to account for the initial sample orientation not being at θ=0° but at $\theta=\omega$. We found that ω values for $B_{3g,\parallel}$ and $B_{3g,\perp}$ differ by approximately 7°, which is well within experimental errors caused by inaccuracies in sample rotation, laser and detector inconsistencies over long experiment times, imperfect polarizations, and baseline inaccuracies. We then took the average ω and offset all subsequent polar plots accordingly to ensure consistency in our analysis.

We further identified the remaining Raman peaks (in Fig. 1c) by observing from the spectra that four of the peaks (including the B_{3g} peak) are distinguishable even without deconvolution, have a small FWHM of 1nm and position approximately that of the established Raman modes. We fitted the three remaining peaks to the said A_g Raman modes and present the polar plots in Supplementary Fig. 1.

Supplementary Note 2. Reflection spectra for samples on Si/SiO_2 for a full *θ* rotation cycle.

Samples for reflection and transmission were grown/ exfoliated on non-absorbing fused SiO_2 substrates to allow for better representation of the optical behavior of the samples through the R and T graphs and the presence of Fabry–Pérot interference modes. Samples were also grown on Si/SiO_2 substrates (Supplementary Fig. 2) to eliminate the possibility of light transmitting through the substrate after passing through the samples to generate similar Fabry–Pérot modes, ensuring that our observations are mostly governed by the samples' optical response.

Reflection measurements were carried out using the same configuration as the PL measurements, while using white light as the light source. We conducted the experiment without a second polarizer because we are only interested in observing the selectivity of the valleys for the absorption of polarized light across the visible-near IR range.

The processed R and T spectra for absorption coefficient and band gap determination are presented in Fig. 3.

Supplementary Fig. 2 shows that under the orientation where the armchair direction is aligned to the axis of polarization, the Fabry–Pérot modes begin to appear at wavelengths longer than 775nm. More accurately, this signifies that the material starts to absorb light at wavelengths shorter 775nm and hence the Fabry–Pérot modes cease to occur because the reflection intensity is solely based on the reflection off the surface of the material, which remains relatively constant barring some slight dispersion-induced effects. This selective absorption is attributed to the ΓY valley.

Increasing the angle between the polarization and material symmetry axes, we see that these Fabry–Pérot modes are dampened but the peak positions between 775nm and 950nm do not shift. This agrees with our understanding that light with wavelength longer than 775nm are now attenuated because the portion of light with **E**-field parallel to the zigzag direction can now be absorbed at the ΓX valley, and hence there is a seemingly attenuated amount of light available for Fabry–Pérot oscillations.

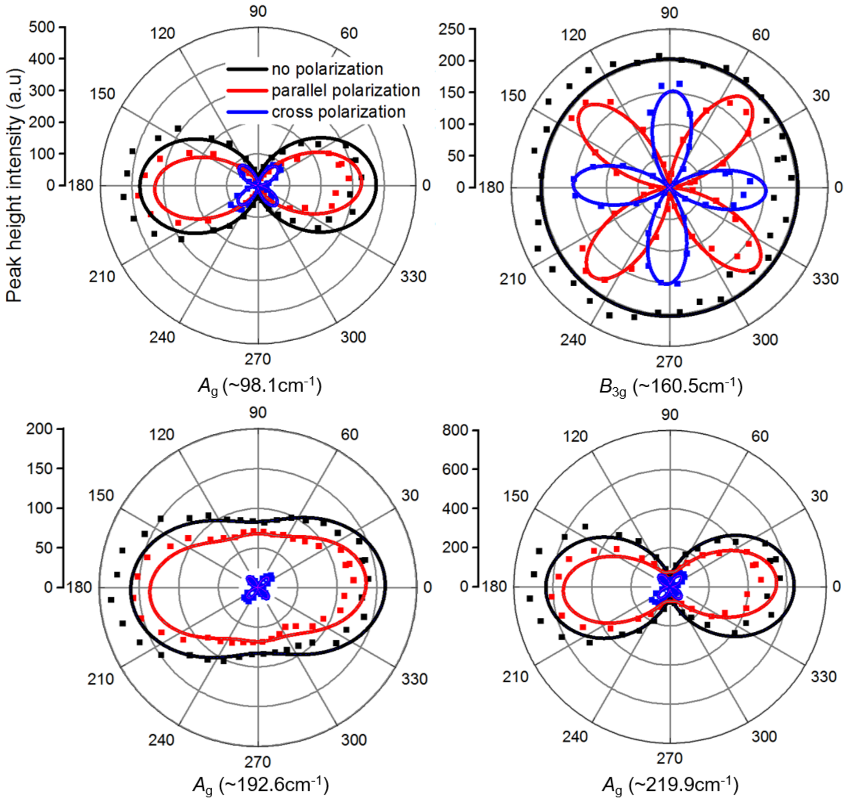

Supplementary Fig. 1: Raman polar plots. Four polar plots of peak height intensity variations in Raman peaks assigned to either A_g or B_{3g} modes. The intensity variations follow the trend in published results and allowed us to have a rigorous derivation of the material axes directions.

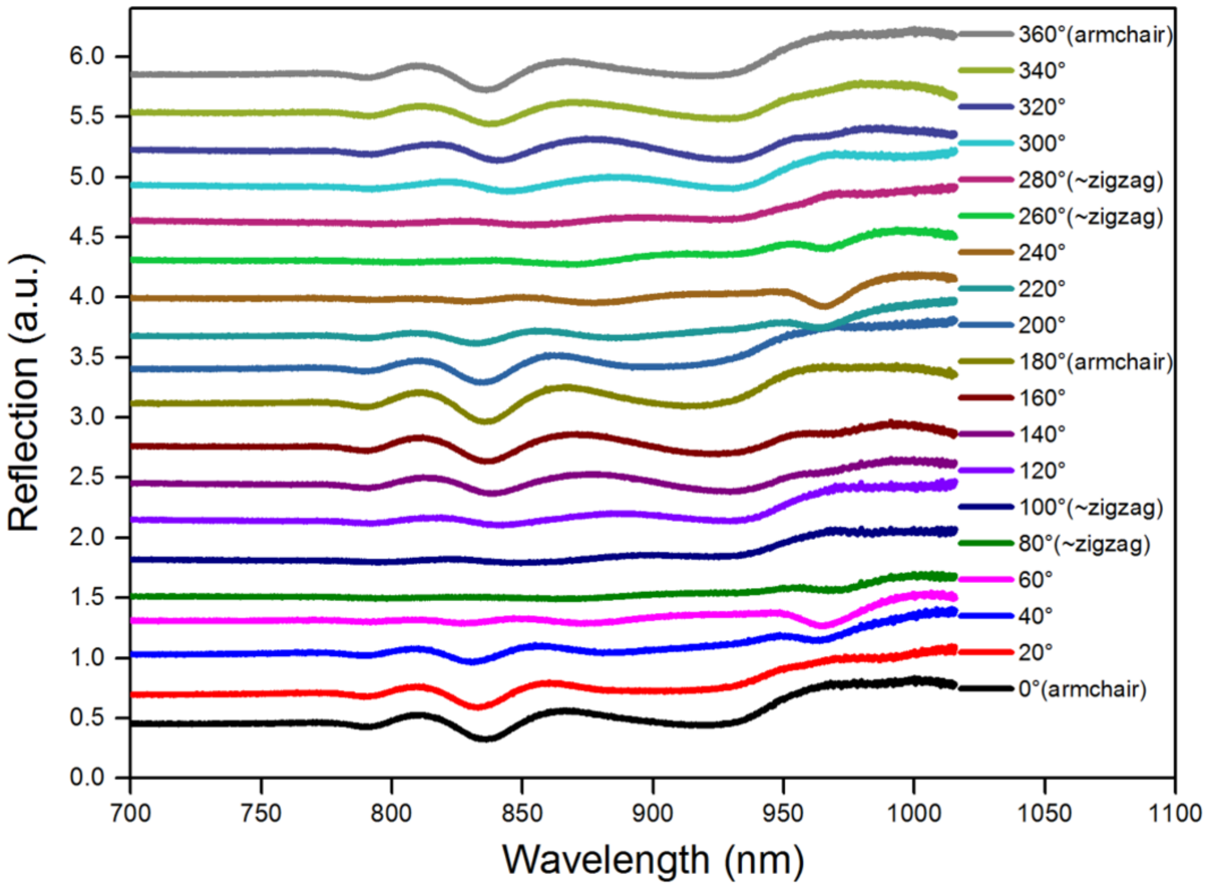

Supplementary Fig. 2: Reflection spectra of SnS with sample rotated every 20° for a full *θ* rotation. Results clearly show that the onset of Fabry–Pérot oscillations for all θ values is at 775nm. At angles away from the armchair axis, the onset of the oscillations remain unchanged while the oscillations are dampened. When the polarization is aligned to the zigzag direction, the oscillations are fully dampened (presumably because absorption at 775nm is no longer allowed) and another onset of Fabry–Pérot oscillations is shown to be 950nm. These two onsets of oscillations can be assigned to the two non-degenerate band gaps in our system that show valley behavior.

References

1. Xia J. et al. Physical vapor deposition synthesis of two-dimensional orthorhombic SnS flakes with strong angle/temperature-dependent Raman responses. Nanoscale 8, 2063–2070 (2016).
